# Genomic Analysis Reveals the Fast-Growing Trait and Improvement Potential for Stress Resistance in the Elite Poplar Variety *Populus* × *euramericana* ‘Bofeng 3’

**DOI:** 10.3390/ijms26125526

**Published:** 2025-06-09

**Authors:** Shanchen Zhong, Weixi Zhang, Changjun Ding, Zhengsai Yuan, Le Shen, Bingyu Zhang, Yanguang Chu, Xiaohua Su

**Affiliations:** 1 State Key Laboratory of Tree Genetics and Breeding, Research Institute of Forestry, Chinese Academy of Forestry, Beijing 100091, China; zhong_shanchen@126.com (S.Z.);; 2 Key Laboratory of Tree Breeding and Cultivation of State Forestry and Grassland Administration, Research Institute of Forestry, Chinese Academy of Forestry, Beijing 100091, China; 3 Jiangxi Provincial Key Laboratory of Improved Variety Breeding and Efficient Utilization of Native Tree Species, Institute of Biological Resources, Jiangxi Academy of Sciences, Nanchang 330046, China; 4 Co-Innovation Center for Sustainable Forestry in Southern China, Nanjing Forestry University, Nanjing 210037, China

**Keywords:** *Populus* × *euramericana* ‘Bofeng 3’, genome, transcriptome, comparative genomics, drought tolerances, salt resistance

## Abstract

Enhancing stress tolerance represents a critical objective in the genetic improvement of poplar trees. *Populus* × *euramericana* ‘Bofeng 3’ is a nationally certified elite poplar variety that was approved as a premium pulpwood variety for the southern area of Northeastern China. This variety grows quickly, has good yield, and resists frost; however, its weaker drought and salt tolerance limits its broader use in diverse environments. The aim of this study is to understand the genetic basis of the fast growth and stress-adaptation traits of this variety and to provide support for future molecular breeding efforts. We present a chromosome-scale genome assembly of *Populus* × *euramericana* ‘Bofeng 3’, totaling 445.53 Mb, of which with 90.39% is anchored to 19 chromosomes, containing 33,309 protein-coding genes and 45.36% repetitive elements. Comparative genomics showed that ‘Bofeng 3’ has expanded gene families related to photosynthesis and metabolism, and contracted families involved in stress responses, distinguishing it from other *Populus* species. Under drought (9137 leaf, 9403 root differentially expressed genes (DEGs)) and salt stress (2840 leaf, 3807 root DEGs), trend analysis revealed specific expression patterns. Several unique and expanded genes, including those for photosynthetic proteins, peroxidases, gamma-aminobutyric acid metabolism, and disease resistance, showed stress-responsive trends. Weighted gene co-expression network analysis identified five modules (three positive, two negative) that significantly correlated with photosynthetic traits, highlighting key candidates such as bZIP transcription factors and auxin/indole acetic acid genes. This study determined the genetic basis underlying the rapid growth traits of *Populus* × *euramericana* ‘Bofeng 3’, while providing genomic resources to establish a robust foundation for future gene editing and molecular breeding studies, including critical candidate genetic resources for developing superior drought- and salt-tolerant poplar varieties via targeted genome editing technologies.

## 1. Introduction

*Populus* spp. (poplar) is a genus used globally for significant fast-growing plantations that comprises approximately 30 species and is widely distributed across the Northern Hemisphere. Among them, the interspecific hybrid *Populus* × *euramericana* (Dode) Guinier, derived from the *Aigeiros* section species, *P. deltoides* Marshall (eastern cottonwood), and *P. nigra* Linn (black poplar), shows good growth performance in subtropical and warm temperate regions of China and serves as a dominant plantation variety, while also playing a critical role in global poplar cultivation [1,2]. The elite variety *P.* × *euramericana* ‘Bofeng 3’, developed by the Research Institute of Forestry, Chinese Academy of Forestry (National Certification No. S-SV-PE-003-2017), originates from a maternal parent of *P. deltoides* intraspecific hybrid ‘Henan 65’ (*P. deltoides* cl. ‘55/65’ × *P. deltoides* cl. ‘2KEN8’) and a paternal parent of *P. nigra* intraspecific hybrid (*P. nigra* ‘Brummen’ × *P. nigra* ‘Piccarolo’). This short-rotation industrial timber variety exhibits rapid growth, high productivity, and frost tolerance, making it ideal for pulp, timber, and biomass energy production, with extensive cultivation in provinces including Liaoning and Shandong in China.

Recent advances in high-throughput sequencing technologies have promoted a shift from traditional phenotype-based selection to genotype-driven molecular breeding in forest trees. *Populus* has become a model species for tree genetic and breeding research because of its relatively small genome, rapid growth, easy vegetative propagation, and well-established genetic transformation systems. The availability of whole-genome sequences enables the identification of functional genes, regulatory elements, and molecular markers associated with traits of interest. By integrating genome assemblies with transcriptomic data under stress conditions, researchers can explore gene expression dynamics and identify candidate genes involved in stress responses and growth regulation. Such integrative approaches provide a molecular foundation for genome editing and targeted improvement of elite cultivars like *P*. × *euramericana* ‘Bofeng 3’, with the goal of enhancing stress resilience and maintaining high productivity. Since the first genome release of *P. trichocarpa* Torr. & Gray [3,4], approximately 20 *Populus* genomes have been sequenced, including *P. trichocarpa* [3,4], *P. tremula* Linn. [5,6], *P. tremuloides* Michx. [6], *P. davidiana* Dode. [7], *P. adenopoda* Maxim. [8], *P. euphratica* Oliv. [9,10,11], *P. tomentosa* Carrière. [12], *P. wilsonii* Schneid. [13], *P. lasiocarpa* Oliv. [14], *P. pruinosa* Schrenk. [15], *P. alba* var. *pyramidalis* Bunge. [16], *P. alba* Linn. [17], *P. simonii* Carr. [18], *P. koreana* Rehder [19], *P. qiongdaoensis* T. Hong & P. Luo [20], *P. alba* × *P. berolinensis* [21], *P. deltoides* × *P. euramericana* ‘Nanlin 895’ [22], *P. alba* × *P. tremula* var. glandulosa (clone 84K) [23], *P. deltoides* cultivar I-69 [7], and *P. tremula* × *P. alba* (INRA 717-1B4) [24]. These released genomic datasets provide important toolkits to improve the quality and accelerate selection breeding processes in poplar varieties. However, most sequenced poplar species are natural genotypes with limited adaptability and application ranges. Notably, genomic resources remain scarce for elite poplar varieties that combine high yield, superior quality, rapid growth, broad adaptability (particularly for northern regions), and efficient genetic transformation systems. This critical gap severely hinders the precise molecular improvement of stress-resistant traits in commercially valuable poplar varieties.

Herein, we report the assembly of a high-quality genome sequence for the elite variety *P.* × *euramericana* ‘Bofeng 3’ (BF3) by integrating Illumina Hiseq, PacBio Sequel II/Sequel IIe, PacBio HiFi reads, and High-throughput Chromosome Conformation Capture (Hi-C) technologies. Additionally, transcriptomic sequencing and gene expression analyses identified key drought- and salt-tolerance-associated genes with negative regulatory roles under stress conditions. These findings provide novel insights into the genomic architecture of *P.* × *euramericana* ‘Bofeng 3’ and its molecular mechanisms underlying drought and salinity resistance. This study establishes a foundation to advance precise molecular breeding in poplar through gene editing technologies, enabling targeted genetic improvements to enhance cultivation efficiency in marginal and difficult lands, as well as to elevate the economic viability and practical application potential of this commercially valuable poplar variety.

## 2. Results

### 2.1. Estimation of the Genome Size and Analysis of the Genome Assembly

**Figure 1:**
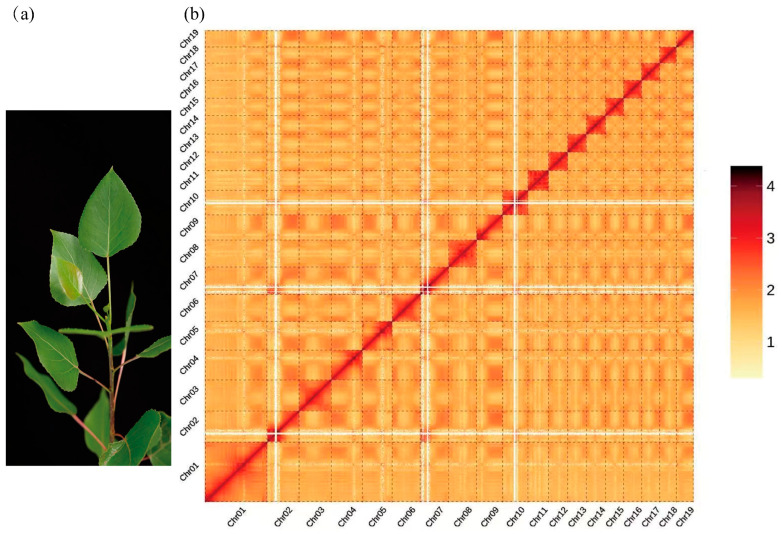
Overview of the *Populus* × *euramericana* ‘Bofeng 3’ genome. (**a**) Morphology of *P.* × *euramericana* ‘Bofeng 3’. (**b**) Hi-C heatmap of *P.* × *euramericana* ‘Bofeng 3’ chromosome interactions.

A kmer17-based survey analysis of the sequencing data (25.00 Gbp) estimated the genome to be of moderate size (471.13 Mbp, adjusted to 462.25 Mbp), with high heterozygosity (2.41%), and a repetitive sequence proportion of 45.51%. Assembly using kmer41 generated scaffolds with a contig N50 of 2979 bp and a total length of 672.16 Mbp, while the scaffold N50 reached 3567 bp, with a total length of 678.58 Mbp (Table 1 and Tables S1–S3). These metrics suggested that the ‘Bofeng 3’ (hereafter referred to as BF3) genome is highly complex. PacBio sequencing yielded 55.00 Gbp of data for the BF3 genome. A total of 30 Gbp of sequencing data (64.90× coverage, based on the survey-adjusted genome size of 462.25 Mbp) (Table S1) was used for de novo assembly. The final assembly produced contigs with a total length of 471.77 Mbp and a contig N50 of 21.71 Mbp (Table 1 and Table S4). The assembly was rigorously evaluated using Benchmarking Universal Single-Copy Orthologs (BUSCO), Core Eukaryotic Genes Mapping Approach (CEGMA), and sequence consistency analyses, demonstrating high genome consistency, completeness, and accuracy (Tables S5–S7). Hi-C data were used to anchor the genome to chromosomes. The final chromosome-level assembly comprised 19 chromosomes (402.72 Mbp anchored) and 827 unanchored sequences, achieving a total genome length of 445.53 Mbp and a chromosome anchoring rate of 90.39% (Table 1, Tables S8 and S9). A genome-wide interaction map revealed strong intra-chromosomal interactions among the 19 chromosomes of BF3, consistent with the expected interaction patterns, further validating the high quality of the Hi-C-assisted assembly (Figure 1).

### 2.2. Genome Annotation

**Figure 2:**
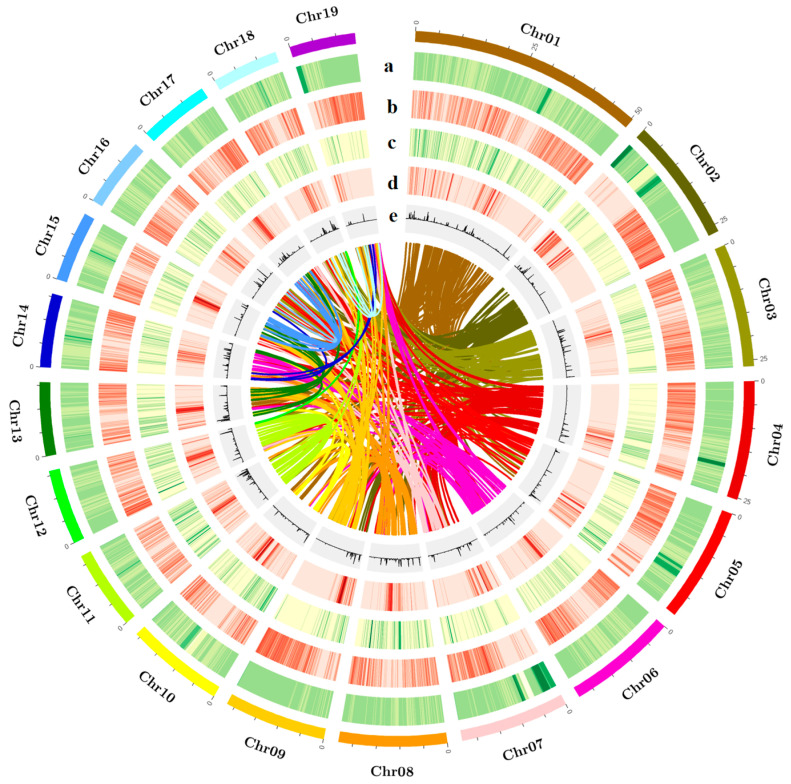
Characterization and synteny of the *P*. × *euramericana* ‘Bofeng 3’ genome. (**a**) The density of Ogre, a family of Gypsy LTR-RTs, (**b**) the density of all Gypsy LTR-RTs, (**c**) the density of all Copia LTR-RTs, (**d**) gene density, and (**e**) histogram of the GC content. The colored lines connect homologous regions.

Repetitive sequences can be classified into two major categories: tandem repeats and interspersed repeats. Tandem repeats include microsatellites and minisatellites, while interspersed repeats, also known as transposable elements (TEs), comprise DNA transposons (transposed via a DNA–DNA mechanism) and retrotransposons (RTs). Common RT categories include long terminal repeats (LTRs), long interspersed nuclear elements (LINEs), and short interspersed nuclear elements (SINEs). By integrating de novo predicted repetitive sequences with the homologous repeat database Repbase, we annotated repeats in the BF3 genome using RepeatMasker. The results showed that 45.36% of the BF3 genome consists of repetitive sequences, including 19.25% LTRs, 1.91% LINEs, and 1.01% SINEs (Table 1 and Table S10). A total of 33,309 protein-coding genes were predicted in the BF3 genome. Functional annotation of the protein sequences derived from gene structure predictions against known protein databases showed that 98.64% of the genes could be functionally annotated (Figure S1, Tables S11 and S12). Functional annotation of the predicted proteins revealed contributions from major public databases to 32,855 genes (98.64%), including non-redundant (NR; 32,750, 98.32%), SwissProt (27,500, 82.56%), Kyoto Encyclopedia of Genes and Genomes (KEGG; 26,355, 79.12%), InterPro (31,795, 95.45%), Pfam (26,397, 79.25%), and Gene Ontology (GO; 21,361, 64.13%) (Table S12) databases. Comparative analysis of the gene structure statistics and genomic features of elements among closely related species showed that the average coding sequence (CDS) lengths for BF3, *P. nigra*, *P. deltoides*, and *P. trichocarpa* were 1236.07 bp, 1399.87 bp, 1246.73 bp, and 1393.60 bp, respectively. The average numbers of exons per gene were 5.22, 5.67, 5.26, and 5.67, respectively (Table S13, Figures S2 and S3). The genome contained 17,645 non-coding RNAs, including 7062 transfer RNAs (tRNAs), 4412 ribosomal RNAs (rRNAs), 535 small nuclear RNAs (snRNAs), and 689 microRNAs (miRNAs) (Table S14). A comprehensive genome assembly and annotation overview was visualized using Circos-0.69-9 software (http://circos.ca/software/) to highlight genomic features (Figure 2).

### 2.3. Comparative Genomic Analysis

**Figure 3:**
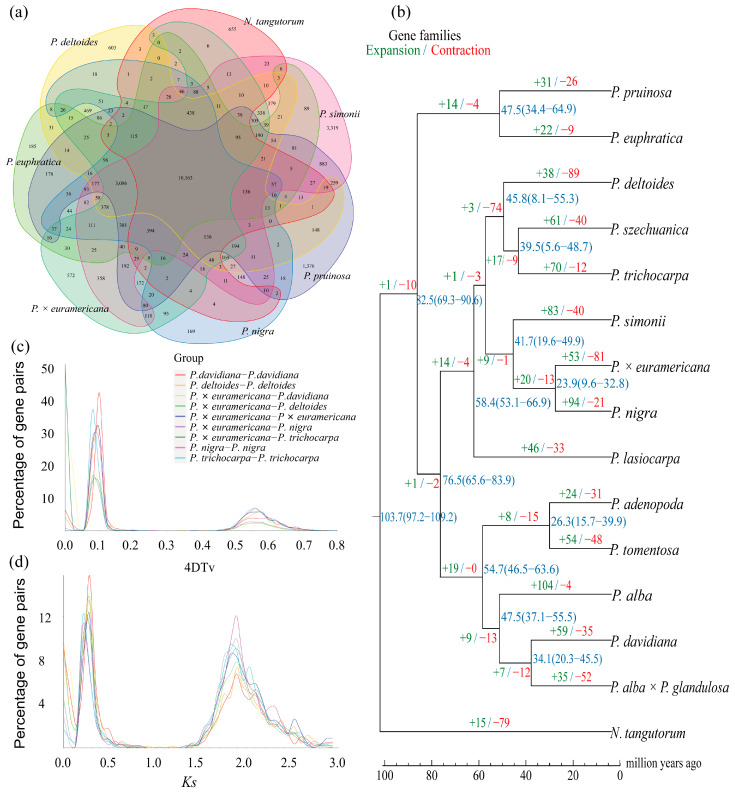
(**a**) The Venn diagram represents the shared and unique gene families. The poplars shown in the picture include *P.* × *euramericana* ‘Bofeng 3’, *P. nigra*, *P. deltoides*, *P. euphratica*, *P. simonii*, and *P. pruinosa*, and the outgroup specie *N. tangutorum*. (**b**) Phylogenetic relationship. Expansion/ contraction of gene families and estimation of divergence time. Blue numbers indicate the divergence time on the nodes, with the confidence range in parentheses. (**c**) The distribution of 4DTV. (**d**) The distribution of *Ks*.

**Figure 4:**
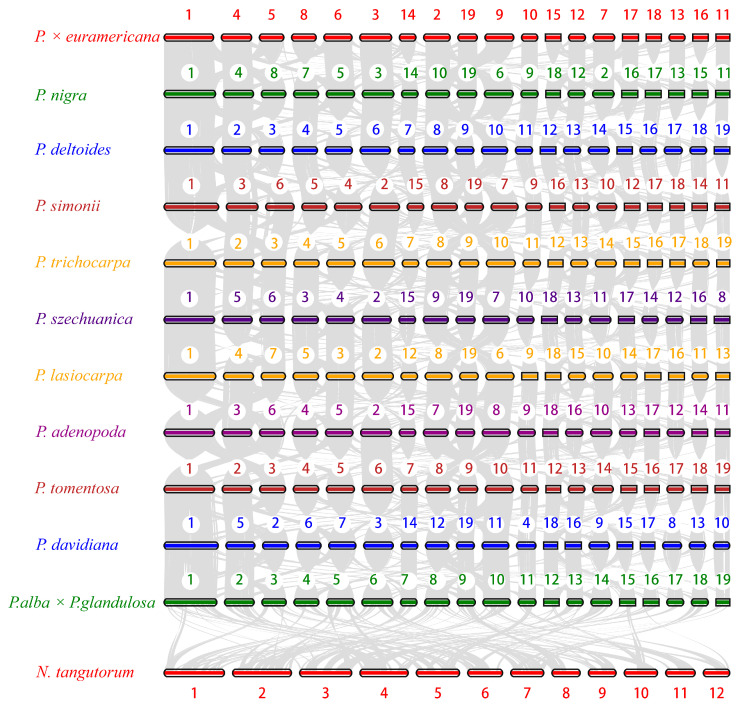
Syntenic plots between *P.* × *euramericana* ‘Bofeng 3’ and other examined species. Syntenic blocks are marked using gray lines.

To investigate the genomic characteristics of BF3 and explore its shared and divergent features with other species in the *Populus* genus, we selected 13 other representative species from diverse sections of the genus, including *P. nigra*, *P. deltoides*, *P. trichocarpa*, *P. simonii*, *P. szechuanica*, *P. euphratica*, *P. pruinosa*, *P. davidiana*, *P. adenopoda*, *P. tomentosa*, *P. alba*, *P. alba* × *P. glandulosa*, and *P. lasiocarpa*. *Nitraria tangutorum* was used as outgroup species for the comparative analysis (Figure S4). For the 15 species, a total of 33,891 gene families were clustered, with 8595 shared across all species, including 2973 single-copy orthologs. Venn diagram analysis (Figure 3a) revealed 1659 unique gene families in the BF3 genome, which is more than those in *P. deltoides* and *P. nigra*, but fewer than those in *P. euphratica*, *P. pruinosa*, *P. simonii*, and *N. tangutorum*. To determine the phylogenetic relationships among species, a maximum likelihood tree was constructed using *N. tangutorum* as the outgroup (Figure 3b). The 14 *Populus* species diverged into two major clades: one consisting of *P. euphratica* and *P. pruinosa*, indicating their close relationship and early divergence (~82.50 million years ago, Mya), and the other encompassing species in sections *Aigeiros* (including BF3 and its paternal parent *P. nigra*), *Leuce* (*P. adenopoda*, *P. tomentosa*, *P. alba*, *P. davidiana*, and *P. alba* × *P. glandulosa*), *Tacamahaca* (*P. szechuanica*, *P. trichocarpa*, and *P. simonii*), and *Leucoides* (*P. lasiocarpa*). Divergence time estimation based on fossil calibrations revealed that the parental species of BF3 (*P. nigra* and *P. deltoides*) diverged from a common ancestor ~53.5 Mya. Gene family expansion/contraction analysis identified 33,886 gene families for the most recent common ancestor (MRCA). A total of 53 expanded and 81 contracted families were determined in BF3, compared with 94 expanded and 21 contracted families in the paternal parent *P. nigra* (Figure 3b). Synonymous substitution analysis of four-fold degenerate (4DTv) sites and Ks distributions for five poplar species revealed two peaks corresponding to whole-genome duplication events (Figure 3c,d): an ancient core eudicot γ triplication (*Ks* ≈ 0.28) and a Salicaceae-specific duplication (*Ks* ≈ 1.89). Left-shifted peaks in the 4DTv/*Ks* plots suggested recent small-scale duplications and ongoing speciation. Collinearity analysis confirmed strong genomic synteny between BF3 and its parental species (*P. nigra* and *P. deltoides*; Figure 4 and Figure S5), consistent with its genetic background. This integrated analysis provides insights into the evolutionary history and genomic diversification within *Populus*, highlighting the unique genomic architecture of BF3 and its phylogenetic context.

Compared with other poplar cultivars, BF3 exhibits high yield and rapid growth, and genes specific to BF3 are primarily associated with biological functions including plant metabolic processes, photosynthesis, and redox regulation. The GO enrichment analysis (Figure S6) revealed that these genes are predominantly enriched in the following four functional categories: (1) Cellular metabolism and substance transport (e.g., cellular metabolic process, small molecule metabolic process, organonitrogen compound metabolic process, carbohydrate derivative metabolic process, nucleotide metabolic process, nucleobase-containing compound metabolic process, and nitrogen compound metabolic process). (2) Energy metabolism and photosynthesis (e.g., photosynthesis, light reactions, electron carrier activity, oxidoreductase activity, phosphorylation, and phosphate-containing compound metabolic process). (3) Aromatic and heterocyclic compound metabolism (e.g., cellular aromatic compound metabolic process, heterocycle metabolic process, and organic cyclic compound metabolic process). (4) Cellular processes and structural components (e.g., membrane, single-organism cellular processes). The partition of these enriched functional categories provides insights into the molecular mechanisms underlying the environmental adaptability and development of BF3. These data further revealed the potential genetic determinants contributing to its distinctive phenotypic traits for growth and stress responses.

The expanded genes of the BF3 genome are primarily associated with metabolic processes, oxidative phosphorylation, and photosynthesis, while the contracted genes are predominantly involved in binding, signaling pathways, and immune responses. Further GO enrichment analysis of the expanded gene families (Figure S7) revealed significant enrichment in cellular process, metabolic process, cellular metabolic process, single-organism process, single-organism cellular process, phosphate-containing compound metabolic process, membrane, oxidoreductase activity, and phosphorylation. The KEGG enrichment analysis demonstrated that the expanded genes were principally enriched in oxidative phosphorylation, photosynthesis, and metabolic pathways (Figure S7).

The GO enrichment analysis of contracted genes showed predominant enrichment in binding, protein binding, heterocyclic compound binding, organic cyclic compound binding, ion binding, nucleotide binding, anion binding, transferase activity, purine ribonucleoside binding, purine ribonucleotide binding, purine ribonucleoside triphosphate binding, protein kinase activity, adenyl ribonucleotide binding, protein phosphorylation, ATP binding, and cell communication (Figure S8). The KEGG enrichment analysis of these genes (Figure S8) identified significant enrichment in multiple pathways, including Toll-like receptor signaling pathway, the nuclear factor kappa B (NF-κB) signaling pathway, Toll and Imd (immune deficiency) signaling pathway, the mitogen-activated protein kinase (MAPK) signaling pathway, herpes simplex virus 1 infection, Epstein–Barr virus infection, the nucleotide-binding and oligomerization domain (NOD)-like receptor signaling pathway, plant–pathogen interaction, the Ras signaling pathway, gap junctions, pathogenic *Escherichia coli* infection, MAPK signaling pathway—plant, phagosome function, and cyanoamino acid metabolism.

The positively selected genes in the BF3 genome are enriched in nucleic acid metabolism, homologous recombination, purine metabolism, and multiple signaling pathways. The GO enrichment analysis of the positively selected genes in BF3, *P. nigra*, *P. deltoides*, *P. euphratica*, and *P. pruinosa* showed enrichment in nucleic acid metabolic process, cellular aromatic compound metabolic process, heterocycle metabolic process, nucleobase-containing compound metabolic process, organic cyclic compound metabolic process, nitrogen compound metabolic process, DNA metabolic process, cellular nitrogen compound metabolic process, and RNA metabolic process. The KEGG enrichment results indicated enrichment in Spliceosome, Homologous recombination, Huntington disease, RNA polymerase, Pentose and glucuronate interconversions, non-homologous end-joining, T-helper (Th)1 and Th2 cell differentiation, Lipopolysaccharide biosynthesis, Purine metabolism, and Base excision repair.

The GO enrichment analysis of positively selected genes in BF3, *P. nigra*, *P. deltoides*, *P. euphratica*, *P. pruinosa*, and *P. trichocarpa* showed enrichment in nucleic acid metabolic process, DNA metabolic process, cellular aromatic compound metabolic process, nitrogen compound metabolic process, heterocycle metabolic process, organic cyclic compound metabolic process, nucleobase-containing compound metabolic process, cellular response to stress, cellular nitrogen compound metabolic process, and RNA metabolic process. The KEGG enrichment results revealed enrichment in Spliceosome, Homologous recombination, Purine metabolism, Base excision repair, Carbon fixation pathways in prokaryotes, Ubiquinone and other terpenoid–quinone biosynthesis, Biosynthesis of siderophore group nonribosomal peptides, the p53 signaling pathway, Thyroid cancer, Mineral absorption, and Soluble N-ethylmaleimide-sensitive factor-activating protein receptor (SNARE) interactions in vesicular transport.

### 2.4. Transcriptome Analysis and Screening of Genes Related to Drought and Salt Stress Tolerance

**Figure 5:**
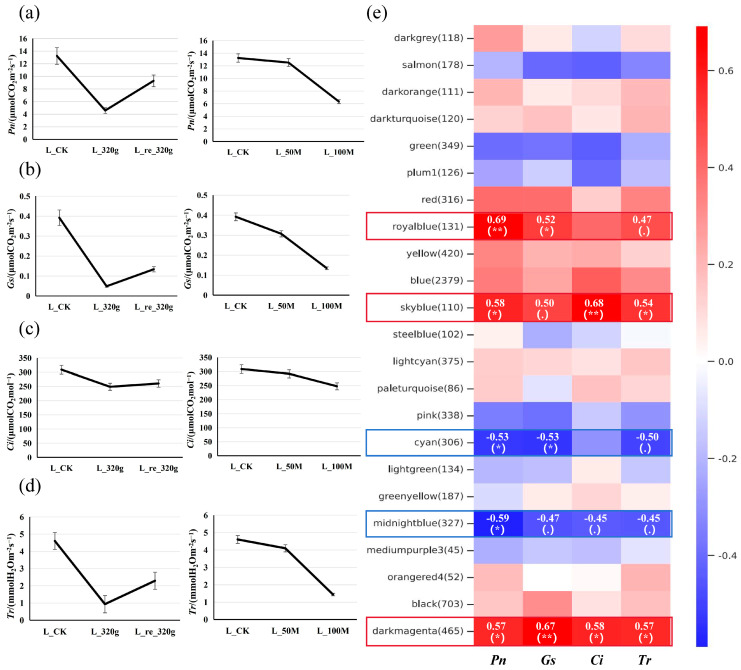
(**a**–**d**) Changes in photosynthetic parameters of potted seedlings under drought/salt stress. L_CK, L_320g, and L_re_320g are the leaves of the control group, those dried for 2 weeks, and those rehydrated for 24 h, respectively; L_50M and L_100M are the leaves of plants treated with 50 mmol·L^−1^ and 100 mmol·L^−1^ salt for 2 weeks, respectively. (**e**) Module–trait association analysis. The heatmap displays Pearson correlation coefficients between module eigengenes and external traits, with statistical significance denoted as follows: * *p* < 0.05 (significant), ** *p* < 0.01 (highly significant), and for 0.05 ≤ *p* < 0.1 (marginally significant).

After drought stress treatment (Figure S9), measurements of photosynthetic parameters in each line revealed a decrease in the net photosynthetic rate (*Pn*), stomatal conductance (*Gs*), intercellular CO_2_ concentration (*Ci*), and transpiration rate (*Tr*), which slightly increased after rewatering. The results of the salt stress treatment showed that a 50 mmol·L^−1^ NaCl treatment for two weeks led to a slight decrease in *Pn*, *Gs*, *Ci*, and *Tr*, while a 100 mmol·L^−1^ NaCl treatment for two weeks resulted in more pronounced decreases (Figure 5a–d). Transcriptome sequencing was performed on the leaves and roots of BF3, with three biological replicates for each sample. The results indicated promising sequencing quality and good alignment of the reads with the reference genome (Tables S15 and S16).

Trend analysis performed on the differentially expressed genes (DEGs) in leaf and root tissues under drought/salt stress treatments allowed for us to distinguish the expression levels of species-specific and expanded genes identified through comparative genomic analysis under stress conditions. Such analysis identified genes that exhibited an expression pattern of initial decrease followed by an increase with drought treatment and rewatering, as well as genes that showed a decreasing expression trend with increasing salt concentration (Figure S10). In the drought stress experiment, 6075 and 6925 genes meeting the above criteria were screened from the leaf and root transcriptome data, respectively. In the salt stress experiment, 3143 and 3364 genes were screened from the leaf and root transcriptome data, respectively. Comparative genomic analysis identified that the species-specific genes that fitted this trend were related to chloroplasts (Poeur.09G01033), disease resistance protein (Poeur.10G00838), and glutamate decarboxylase (Poeur.13G01103). Glutamate decarboxylase participates in the synthesis and partition of gamma aminobutyric acid (GABA) in mitochondria, which regulates plant carbon and nitrogen metabolism. The expanded genes that fitted this trend comprised peroxidase-related genes (Poeur.02G01752, Poeur.03G02312), Hsp90/histidine kinase-related genes (Poeur.01G01324, Poeur.03G02312), photosynthetic reaction center protein genes (Poeur.00G01145, Poeur.00G00303), proton-conducting membrane transporter-related genes (Poeur.00G01282, Poeur.00G02010), disease resistance protein-related genes (Poeur.12G00325, Poeur.15G00090), and a salt stress response/antifungal-related gene (Poeur.10G00184). The expression of these genes might play important roles in the response to stress. With respect to the genes that showed an initial increase in expression followed by a decrease with drought treatment and rewatering, 9137 and 9403 genes were screened from the leaf and root transcriptome data, respectively. For genes that displayed an increasing expression trend with increasing salt concentration, 2840 and 3807 genes were screened from the leaf and root transcriptome data, respectively. Species-specific genes that fitted this trend were a transmembrane transporter-related gene (Poeur.01G00253), glutathione S-transferase-related genes (Poeur.01G00253, Poeur.09G00458, Poeur.09G00678), and tRNA intron endonuclease-related genes (Poeur.07G00966, Poeur.07G01571). Expanded genes that fitted this trend were peroxidase-related genes (Poeur.02G01758, Poeur.02G01759, Poeur.02G01762, Poeur.02G0176, Poeur_02G017403) and an Hsp90 protein/histidine kinase-related gene (Poeur.08G00566).

In addition, weighted gene co-expression network analysis (WGCNA) was performed on the DEGs combined with the data on the photosynthetic parameters (Figure 5e). WGCNA categorizes genes into distinct modules based on their expression patterns across different samples, with each module comprising a cluster of highly correlated genes that exhibit strong co-expression relationships. In the module–trait association analysis, the expression levels of the sky blue module (110 genes), royal blue module (131 genes), and dark magenta module (465 genes) showed positive correlations with photosynthetic parameters, while the expression levels of the cyan module (306 genes) and midnight blue module (327 genes) exhibited negative correlations with photosynthetic parameters. The relationships among the top 50 genes in each module are presented in Figure S11. Trend analysis identified genes from these modules that showed decreased expression initially and then increased expression with drought–rewatering treatment, and that showed decreased expression as the salt treatment concentration increased, which comprised cytochrome P450 genes (Poeur.10G01108, Poeur.12G00613, Poeur.07G01321, etc.) and enhanced disease susceptibility/pathogenesis-related protein genes (Poeur.16G00403, Poeur.19G00701). In contrast, the genes that displayed increased expression initially and then decreased with drought–rewatering treatment and were increased with increased salt treatment concentration comprised bZIP transcription factor-related genes (Poeur.19G00832, Poeur.04G00016, Poeur.08G01073, Poeur.19G00832, Poeur.04G01440, Poeur.06G00012), an auxin/indole acetic acid (AUX/IAA) family gene (Poeur.16G00702), and auxin-responsive protein-related genes (Poeur.14G00116, Poeur.01G03122, Poeur.04G01520).

## 3. Discussion

Genomic analysis revealed that the genome of the elite variety BF3 has a total length of 445.53 Mb, with approximately 90.39% of the sequences being anchored to chromosomes. Compared with other sequenced poplar varieties, the sequencing and assembly quality of the BF3 genome is relatively high, thus providing a reliable foundation for subsequent gene functional annotation and analysis [22,23,24,25]. Annotation of the BF3 genome predicted a total of 33,309 protein-coding genes, with 98.64% of these genes matching known entries in various protein databases, further confirming the reliability of the genome assembly and annotation. Based on annotations from public databases such as GO, KEGG, and InterPro, we found that these genes are closely associated with biological processes such as plant metabolism, photosynthesis, and disease resistance. Notably, genes related to stress resistance, carbon and nitrogen metabolism, and photosynthesis were annotated, such as the glutamate decarboxylase gene (Poeur.13G01103), suggesting that these genes play significant roles in the adaptation to environmental stress for this variety [26]. Additionally, the presence and expansion of plant transposable elements are often associated with genome plasticity, evolution, and adaptive capacity [27]; however, whether the transposable elements and tandem repeats in the BF3 genome have functional significance in the genome evolution and environmental adaptation in this variety remains to be explored.

The rapid growth and strong adaptability of BF3 are largely attributed to its unique genetic background and genomic characteristics. Although natural varieties such as *P. nigra* and *P. deltoides* have limited economic value, their hybrid, BF3, often exhibits superior growth traits and broad adaptability, particularly in terms of rapid growth and stress tolerance. In phylogenetic analysis, *P. deltoides*, *P. nigra*, and BF3 did not cluster within the same clade, with BF3 being closer to *P. nigra* than to *P. deltoides*. This phenomenon might be primarily reflected by the fact that BF3 is a hybrid that inherits genetic material from both *P. nigra* and *P. deltoides*, resulting in genomic heterozygosity that complicates its phylogenetic relationship with its parental species [28]. The closer relationship between BF3 and its paternal parent *P. nigra* might be attributed to the retention of more genetic information from *P. nigra*. Moreover, the significant genomic differences between *P. deltoides* and *P. nigra* might influence their positions on the phylogenetic tree. Although the *Aigeiros* section only includes two natural species (*P. nigra* and *P. deltoides*), they are important hybrid parents and have become primary genetic donor sources of over 90% of cultivated poplar varieties globally, with *P.* × *euramericana* playing the most significant role. Through heterosis, BF3 inherited the stress tolerance of *P. nigra* and the rapid growth potential of *P. deltoides*, achieving fast growth under suitable environmental conditions.

The rapid growth characteristics of BF3 might be closely related to the expression of certain genes, particularly those involved in photosynthesis, metabolic regulation, and plant growth. Its cold tolerance can be linked to the expression patterns of cold-responsive genes. For example, the Hsp90 protein/histidine kinase might play a crucial role under low-temperature stress, as demonstrated by its functions in plant development, stress responses, and disease resistance [29,30]. The expression of these genes in BF3 might enable it to grow better in regions with cold climates. Although BF3 showed strong and rapid growth and cold tolerance, this variety exhibits weaker tolerance to saline–alkali and drought stress conditions, which might be related to the expression patterns of specific genes and expanded gene families under these stress conditions. Genes associated with drought/stress responses might not be as fully activated in BF3 as they are in other stress-tolerant varieties. For instance, transcriptomic data from BF3 revealed changes in the expression of some antioxidant-related genes (e.g., peroxidase genes Poeur.02G01752 and Poeur.03G02312), proton-conducting membrane transporter/aquaporin genes (e.g., Poeur.00G02010), and disease resistance-related genes (e.g., Poeur.10G00838 and Poeur.12G00325) under drought and salt stress; however, the overall magnitude of their expression levels were relatively low. Studies have shown that enhanced expression levels of antioxidant enzymes such as superoxide dismutase (SOD), peroxidase (POD), and catalase (CAT) are crucial for plant stress tolerance under saline–alkali and drought conditions [31,32]. The enhanced expression of aquaporin genes can promote plant adaptation to drought [33], and disease resistance genes play multiple roles in stress responses by regulating plant immunity and enhancing stress tolerance [34]. The weaker performance of BF3 under high salinity or drought conditions might be caused by the insufficient regulation of these genes, thereby affecting its ability to cope with environmental stresses.

Observations of photosynthetic parameters in BF3 under mild stress conditions revealed that drought and salt stress treatments led to a decline in photosynthesis-related parameters. Photosynthesis is a fundamental metabolic process that plants rely on for growth and development. Decreased soil moisture content causes changes in plant photosynthetic indicators, thereby affecting plant growth [35]. Photosynthesis is also a physiological process that is sensitive to salt stress [36]. Under adverse stress conditions (such as drought and salt stress), plants activate a series of genes to maintain their growth and adapt to environmental changes. Through trend analysis of DEGs, we found that the expression of genes related to photosynthesis, carbon and nitrogen metabolism, and disease resistance changed under stress conditions. In particular, genes associated with chloroplast function, and those encoding glutamate decarboxylase (GAD) and disease resistance proteins, exhibited fluctuating expression patterns in response to changes in drought and salt concentrations, suggesting that these genes might play important regulatory roles in plant responses to environmental stress. For instance, the expression of genes encoding photosynthetic reaction center proteins, peroxidases, and cytochrome P450s under stress conditions might help plants to maintain their basic physiological processes in challenging environments [37,38,39]. The expression of these genes might have played a significant role in the fast-growing trait of BF3, supporting rapid growth by enhancing its adaptability to the environment. WGCNA further revealed correlations between the expression of certain genes and photosynthetic parameters. Genes encoding cytochrome P450s, disease resistance proteins, bZip transcription factors, and auxin-related proteins showed expression patterns that coordinated with the trends observed in drought–rewatering treatments and salt concentration gradient treatments, indicating that these genes might play key roles in drought and salt tolerance by regulating plant physiological processes [39,40,41,42]. In addition, peroxidase genes and Hsp90 protein genes might also help plants to maintain a better growth status in adverse environments by improving their stress resistance. Functional annotation of these key genes also provides important information on targets for gene editing and molecular breeding [43]. Among these genes, glutamate decarboxylase genes, peroxidase genes, cytochrome P450 genes, and disease resistance protein genes can represent potential target genes for gene editing studies in this variety. By knocking out or regulating the expression of these genes, the drought and salt tolerance of BF3 is expected to be further enhanced. Furthermore, similar manipulation of disease susceptibility-enhancing and pathogenesis-related protein genes could not only enhance the drought and salt tolerance of BF3, but also potentially improve its disease resistance, resulting in plants with greater stress resistance that can adapt to more diversified environments.

Increasing environmental variability and unsustainable human activities have led to severe ecological challenges, including accumulation of soluble salts in groundwater, increased frequency of natural disasters, soil salinization, and drought stress. These abiotic constraints have negative impacts on plant growth and development, causing substantial losses in agricultural and forestry productivity. Consequently, it has become imperative to breed tree varieties with breakthrough stress-tolerant traits to cope with environmental deterioration and to enhance the utilization of marginal and difficult to cultivate lands. Advances in gene editing technologies provide critical tools for precision molecular breeding, enabling targeted modification of functional genes to rapidly develop novel stress-resistant tree varieties. However, this approach requires a comprehensive understanding of genomic characteristics to identify key genetic information and candidate functional genes with breeding potential [44]. Since the early 21st century, research on genetically modified plants targeting one or two candidate genes has been carried out [45]. Such approaches focus on the precise regulation of specific genes [46]. However, because of the complexity of woody perennial species like poplar, establishing efficient gene editing systems and selecting suitable candidate genes remains a significant challenge [47]. In this study, we aim to go beyond single-gene targeting by providing a comprehensive genomic and transcriptomic framework for BF3, which allows for the identification of multiple functionally relevant gene candidates related to growth and stress responses. However, whether editing these genes can achieve the desired effects without affecting normal plant growth still requires further experimental validation.

It is important to recognize that fast growth and strong stress resistance are often considered conflicting traits in plant development [48]. Fast-growing plants tend to prioritize energy allocation for biomass accumulation over defense mechanisms, which may limit their ability to adapt to adverse conditions such as salinity–alkalinity and drought stress [49]. Although the regulation of certain genes, such as aquaporins and stress-related transcription factors, can enhance tolerance under stress, the application of these genes in molecular breeding must be considered with caution. A key challenge lies in the temporal regulation of these genes: many stress-response genes should only be activated under specific environmental conditions to avoid unnecessary energy consumption during normal growth. Therefore, future gene editing strategies should prioritize the modification of stress-inducible regulatory elements or the development of conditional expression systems, allowing for plants to autonomously activate defense pathways only when needed. By further exploring inducible or tissue-specific promoters, or clustered regularly interspaced short palindromic repeats (CRISPR)-based regulatory elements that allow for gene expression to be modulated only under stress conditions, unintended energy trade-offs could be minimized during normal growth.

Moreover, ensuring suitable growth conditions is essential to maximize the performance of BF3 poplar, especially in arid and semi-arid regions. Cultivation strategies, such as precise irrigation, soil salinity management, and nutrient optimization, can enhance plant vigor and reduce exposure to abiotic stress, thereby supporting both growth and resilience [50]. However, it is important to emphasize that the primary goal of genetic improvement in poplar, including BF3, is to enhance stress tolerance and increase the efficiency of afforestation on marginal or challenging sites. Under such conditions, fast growth is relative and depends largely on the interplay between genetic potential and environmental factors. Therefore, while optimized cultivation practices can improve growth conditions and facilitate gene expression, they should serve as a complementary strategy to genetic improvement, not a substitute. The future development of BF3 poplar should focus on the integration of molecular breeding and silvicultural optimization to maximize performance under adverse conditions.

## 4. Materials and Methods

### 4.1. Plant Material and Genome Sequencing

The newly developed *P.* × *euramericana* ‘Bofeng 3’ (BF3) was used as the study material. Female plants were sampled, and cuttings were cultivated in the greenhouse of the Chinese Academy of Forestry. DNA was extracted from fresh young leaves of the whole plants, newly grown from one-year-old cuttings. The DNA samples were fragmented using a Covaris ultrasonic device (Covaris, Woburn, MA, USA). After library preparation, sequencing was performed using the Illumina HiSeq platform (Illumina Inc., San Diego, CA, USA) for paired-end (PE) sequencing, and long-read sequencing was conducted on the PacBio Sequel II platform (PacBio Inc., Menlo Park, CA, USA) [51,52].

### 4.2. Genome Assembly and Quality Evaluation

Genome assembly was carried out using Hifiasm, which starts from an uncollapsed genome to preserve haplotype information [53,54,55]. The assembly quality was assessed using BUSCO and CEGMA for sequence completeness [56,57], Burrows-Wheeler Aligner (BWA) for sequence consistency [58], and Merqury [59] for sequence accuracy.

### 4.3. Chromosome Assembly Using Hi-C

Hi-C technology was employed to capture genome-wide chromatin interactions. The Hi-C sequencing data underwent preprocessing to obtain clean data, which was then used for chromosome-level assembly [60,61,62].

### 4.4. Genome Annotation

Genome annotation included repetitive sequence annotation, gene structure prediction, and functional annotation. Repetitive sequences were identified using RepeatMasker V4.1.2-p1 and RepeatModeler V2.0.3 [63,64,65]. Gene structures were predicted using both homology-based methods by comparison with the reference sequences of species like *P. deltoides*, *P. nigra*, and *P. trichocarpa*, and de novo methods using Augustus V3.5 [66] and SNAP software (V2013.11.29) [67]. Transcriptional data were integrated, and annotations were refined using EVidenceModeler (EVM V1.1.1) [68] and PASA software V2.0.2 [69]. Functional annotation was performed by comparing the predicted genes with protein databases such as SwissProt, Nr, Pfam, KEGG, and InterPro.

### 4.5. Comparative Genomic Analysis

Fourteen species from different lineages of the genus *Populus* and one outgroup species were selected for comparative genomic analysis (Table S17). Protein sequences from all species were compared using all-vs.-all BLASTP, with a default E-value of 1 × 10^−5^. Gene families were clustered using OrthoMCL V1.4, with an inflation factor of 1.5. Phylogenetic analysis was carried out using single-copy gene families and the PAML 4.9 software package, specifically the MCMCTree tool [70].

Time calibration points were all taken from the TimeTree website (http://www.timetree.org/) [71,72]. The expansion and contraction of gene families were determined using CAFÉ [73]. Protein sequences were aligned using MUSCLE v3.8.31, and the CDSs were aligned accordingly [74]. The 4DTv and *Ks* values were calculated to estimate the timing of duplication events, and the frequency distribution plots of these values were generated [71,75]. Genome duplication was analyzed using MCScanX, which identifies syntenic blocks between genomes based on gene positions and protein sequence similarity [76].

### 4.6. Drought and Salinity Stress Treatment

Three-month-old plants from BF3 cuttings grown in pots were subjected to mild drought/salt stress treatments (the leaves did not exhibit severe wilting throughout the treatment). The plants to be transplanted were treated when they grew to about 50 cm tall. Drought stress was controlled by the soil weighing method for the relative water content (RWC). The control group (CK) was maintained at an RWC of 60%, while the drought treatment group had an RWC of 20%. After two weeks of treatment, rehydration was carried out for 24 h. The salt stress control group (CK) maintained an RWC of 60%, and the treatment groups were treated with 50 mmol·L⁻^1^ and 100 mmol·L⁻^1^ NaCl, respectively. Daily water supplementation was provided to keep the weight consistent with that of the control group, and the treatment lasted for two weeks. During sampling, the *P*n, *G*s, *C*i, and *T*r were recorded. The photosynthetic parameters were measured on functional leaves that were fully unfolded and located at the 3rd to 5th positions from the stem tip downward, and the average values were taken. Fully expanded leaves (the 3rd to 4th leaf from the top) and corresponding root tissues were collected for transcriptome sequencing.

### 4.7. Transcriptome Data Analysis

Each sample contained three biological replicates. The RNA Nano 6000 Assay Kit of the Bioanalyzer 2100 system (Agilent Technologies, Santa Clara, CA, USA) was employed to assess the total amounts and integrity of RNA. After library preparation and cluster generation, transcriptome sequencing was sequenced on the Illumina Novaseq platform. For data analysis, quality control of raw reads, read mapping to the reference genome, and identification of differential gene expression (|log2FC, fold change| ≥ 1 and *p*-value < 0.05) were carried out. The differentially expressed genes were analyzed using DESeq2 v1.22.1 [77]. Subsequently, the clusterProfiler software package was employed to conduct GO functional enrichment analysis and KEGG pathway enrichment analysis on the differential gene sets [78]. The enrichment analysis was based on the hypergeometric distribution principle. The differential gene sets comprised DEGs obtained from the differential significance analysis and annotated to the gene sets in the GO or KEGG databases. The background gene sets were those that underwent differential significance analysis and were annotated to the GO or KEGG databases.

## 5. Conclusions

In this study, we successfully assembled a high-quality genome of BF3, providing a valuable genomic resource for molecular breeding. Comparative genomic analysis revealed that its rapid growth might be related to the expansion of gene families involved in photosynthesis and primary metabolism, while relatively weak stress tolerance could be linked to the contraction and downregulation of specific stress-responsive genes. Transcriptomic profiling under drought and salt stress conditions identified several candidate genes, which may serve as potential targets for genetic improvement. These findings deepen our understanding of the biological characteristics of BF3 and will aid future efforts to enhance its stress resilience through genomic approaches.

## Figures and Tables

**Table 1:** Sequencing, assembly, and annotation statistics.

Genomic Feature	Values
Total genome sequencing data	55 Gbp
Genome size (estimated)	462.25 Mbp
Genome size (assembled)	445.53 Mbp
GC content	33.91%
Contig N50 of the assembly	21,715,612 bp
Contig total length	471,766,084 bp
Mapping rate	94.54%
BUSCO	99.1%
CEGMA	95.56%
Repeat sequences of genome	45.36%
Number of protein-coding genes	33,309
Functional annotated genes	32,855
Number of non-coding RNAs	17,645

## Data Availability

The data that support the findings of this study are available from the corresponding author upon reasonable request.

## Supplementary Materials

The following supporting information can be downloaded at: https://www.mdpi.com/article/10.3390/ijms26125526/s1.
