# Supplementary material for: Genomic Analysis Reveals the Fast-Growing Trait and Improvement Potential for Stress Resistance in the Elite Poplar Variety Populus × euramericana ‘Bofeng 3’
Source: Int J Mol Sci. 2025 Jun 9;26(12):5526. doi: 10.3390/ijms26125526 (PMC12192602; doi:10.3390/ijms26125526)
Supplement: Supplementary file 1 [file ijms-26-05526-s001.zip › Supplementary Figures.pdf]

Supplementary Figures S1–S11

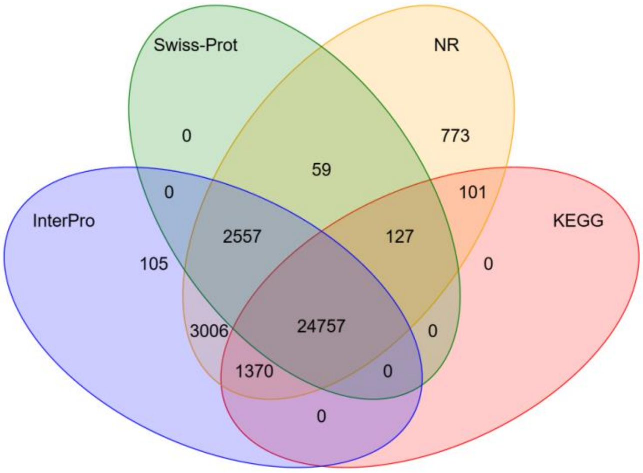

Figure S1. Venn diagram of gene functional annotation for BF3 using four databases.

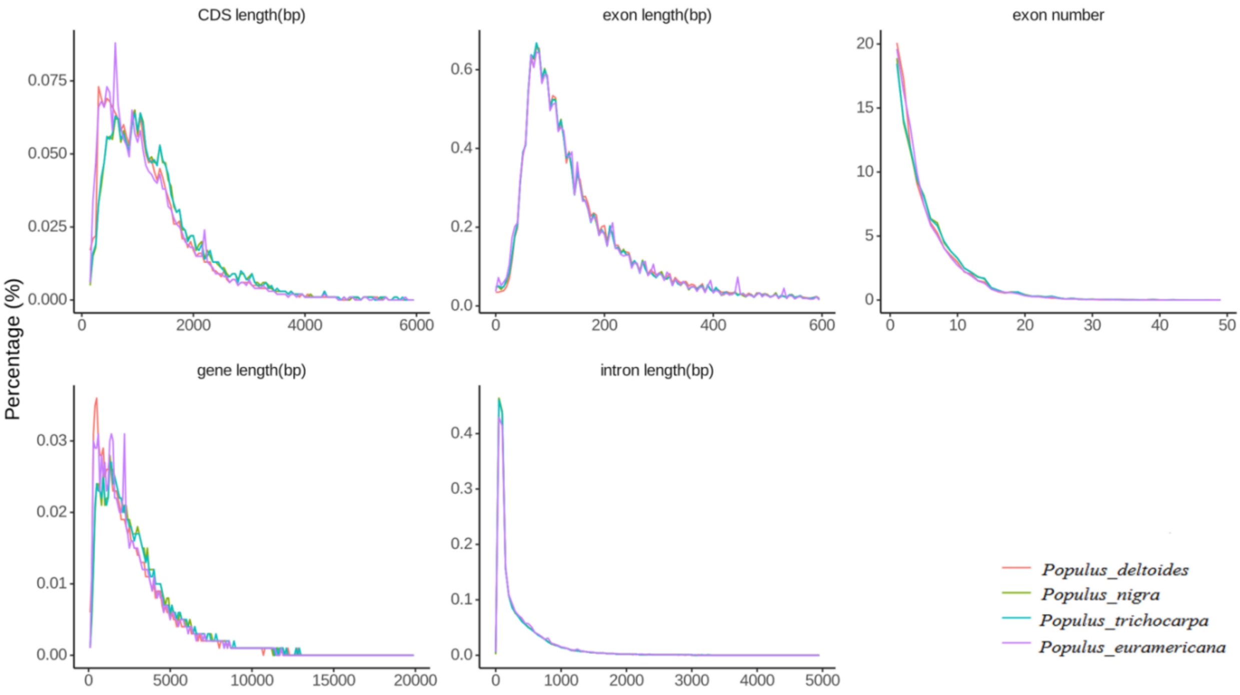

Figure S2. Comparison of the elements of related species.

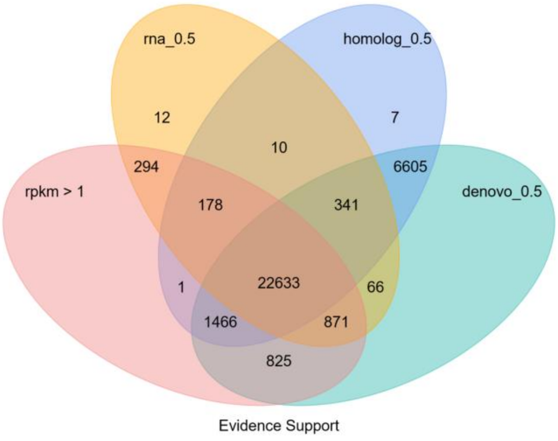

Figure S3. Venn diagram of gene sets found in the BF3 genome. *De novo*, EVM integrated genes supported by *De novo* prediction; Homolog, genes supported by homologous prediction; RNA, genes supported by RNA-seq. The number indicates the gene number.

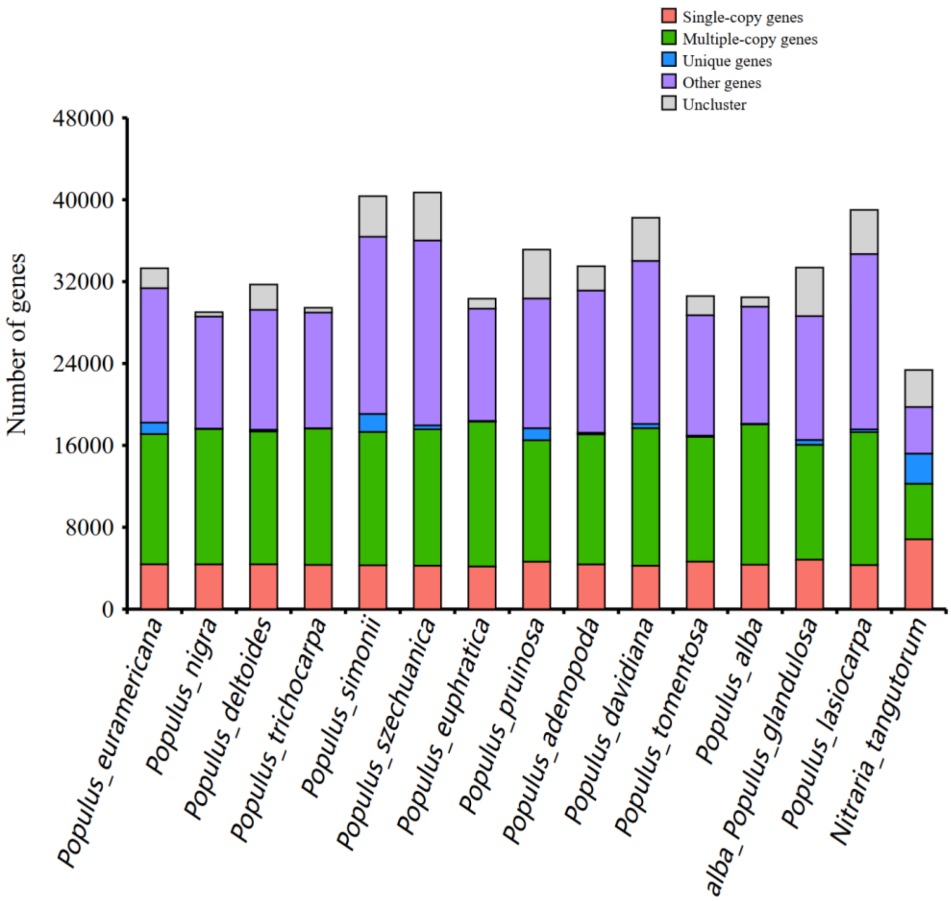

Figure S4. Distribution of gene numbers and family sizes in BF3 and 14 other representative species.

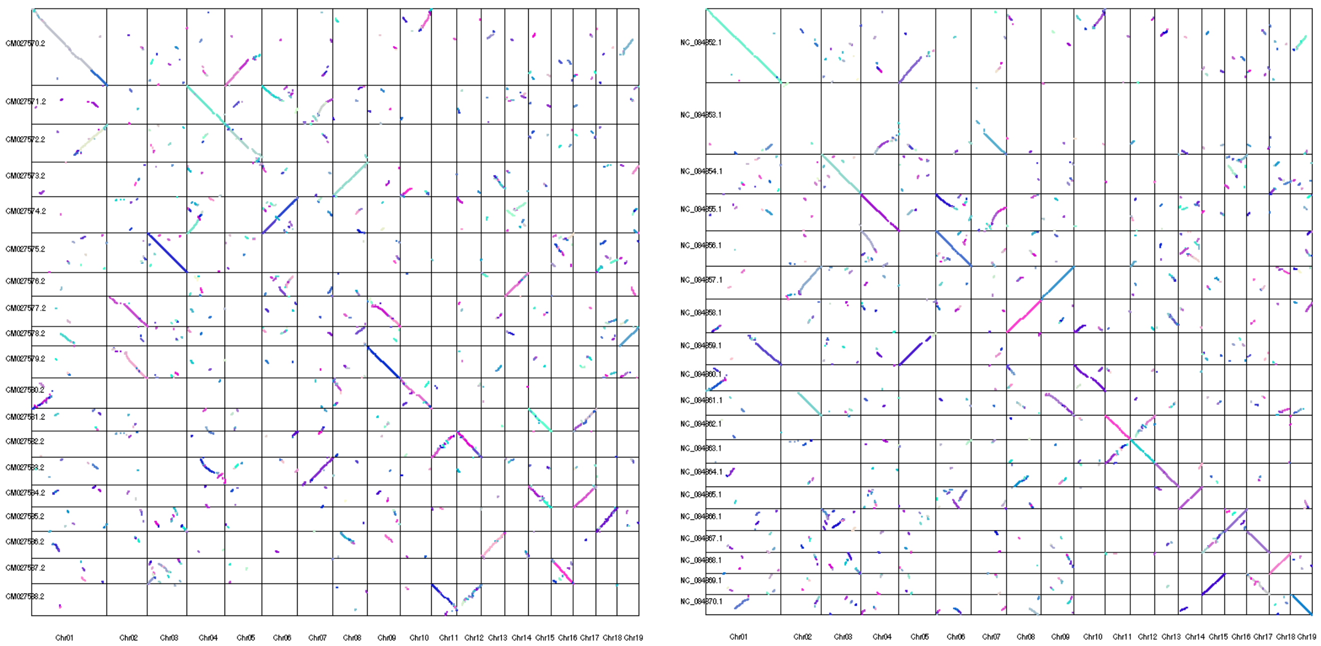

Figure S5. Dotplot analysis of the homology between BF3 and *P. deltoides* (left)/*P. nigra* (right) genomes.

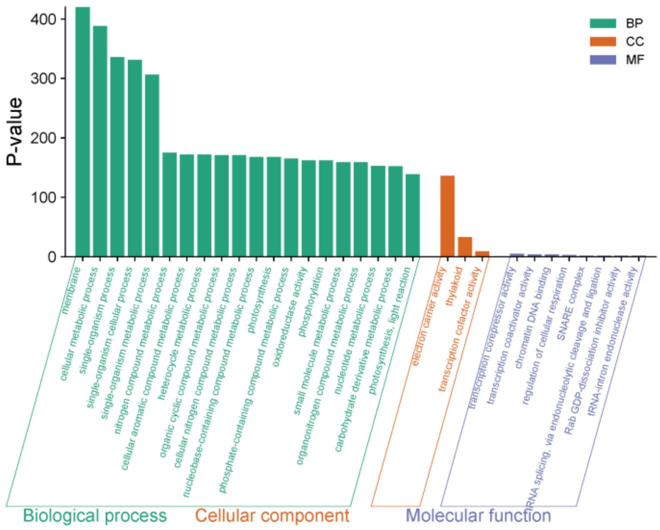

Figure S6. GO enrichment results for exclusive genes.

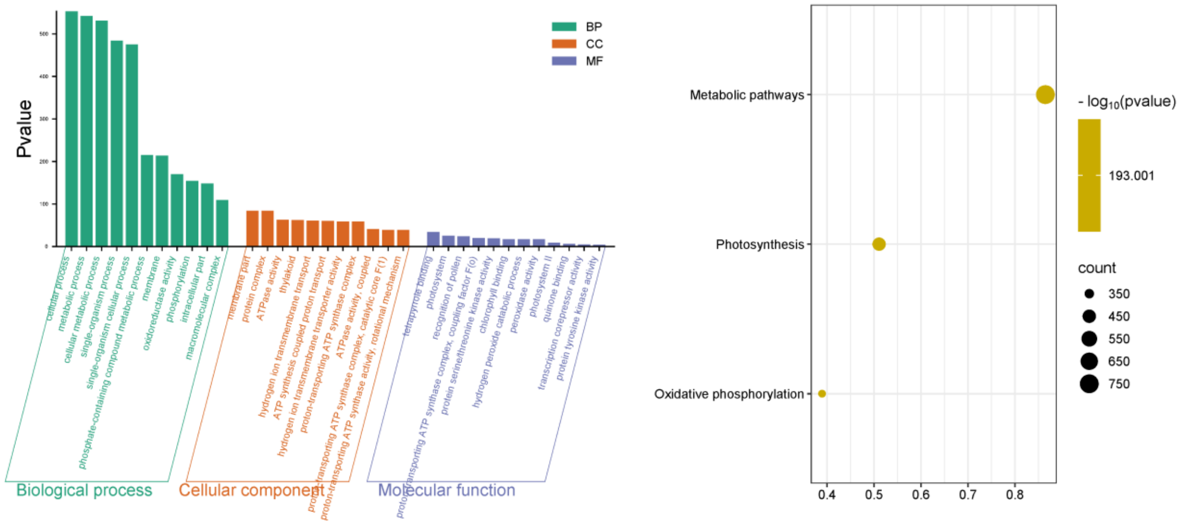

Figure S7. GO (left) and KEGG (right) enrichment results for expanded gene families.

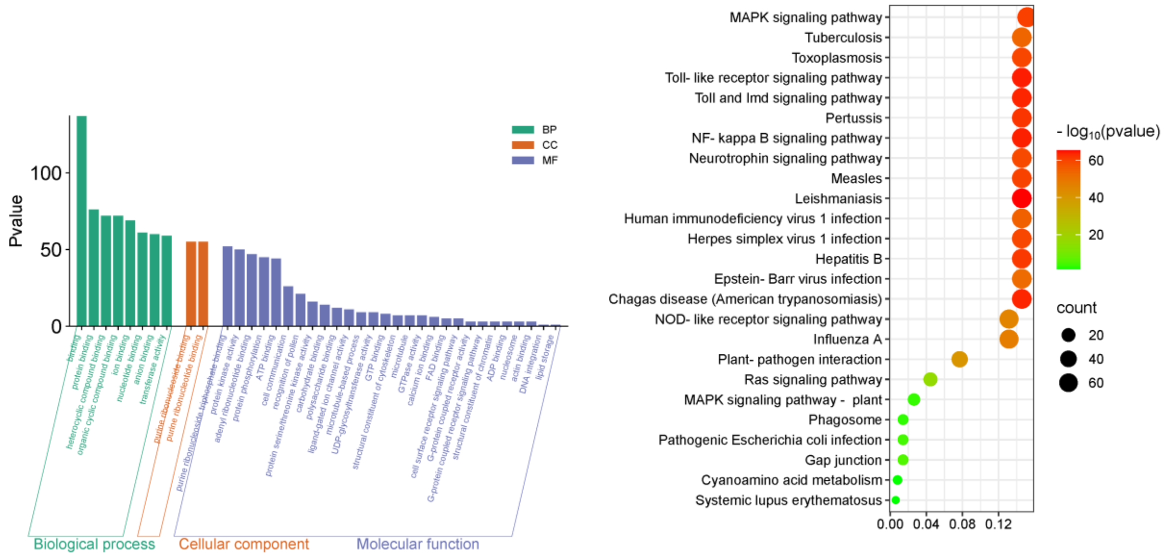

Figure S8. GO(left) and KEGG(right) enrichment results for contracted gene families.

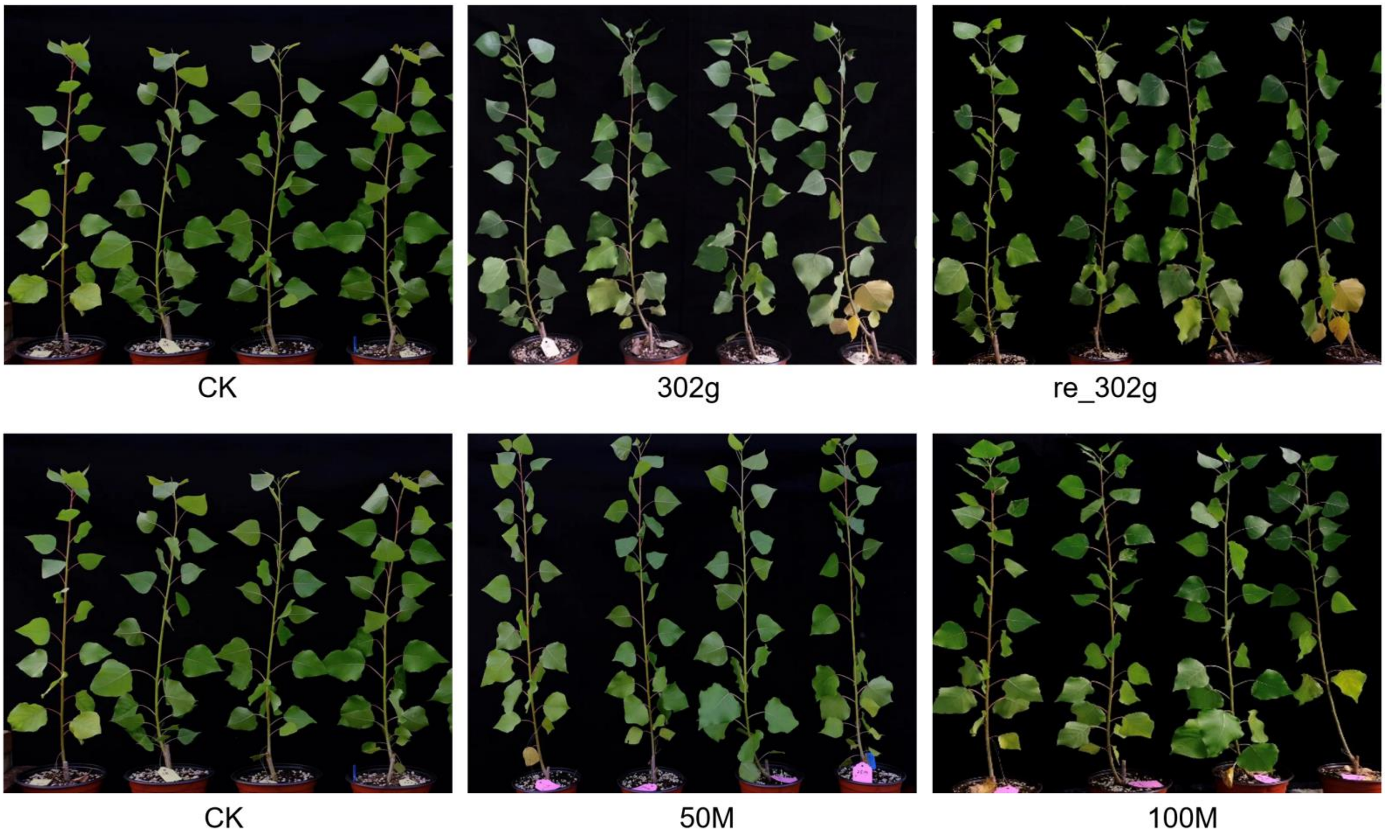

Figure S9. Morphology after drought/salt stress treatment. CK, 320 g and re_320 g indicate the leaves of control group, dried for 2 weeks and rehydrated for 24 hours, respectively; 50M and 100M are the leaves of plants treated with 50 mmol·L^{-1} and 100 mmol·L^{-1} salt for 2 weeks, respectively.

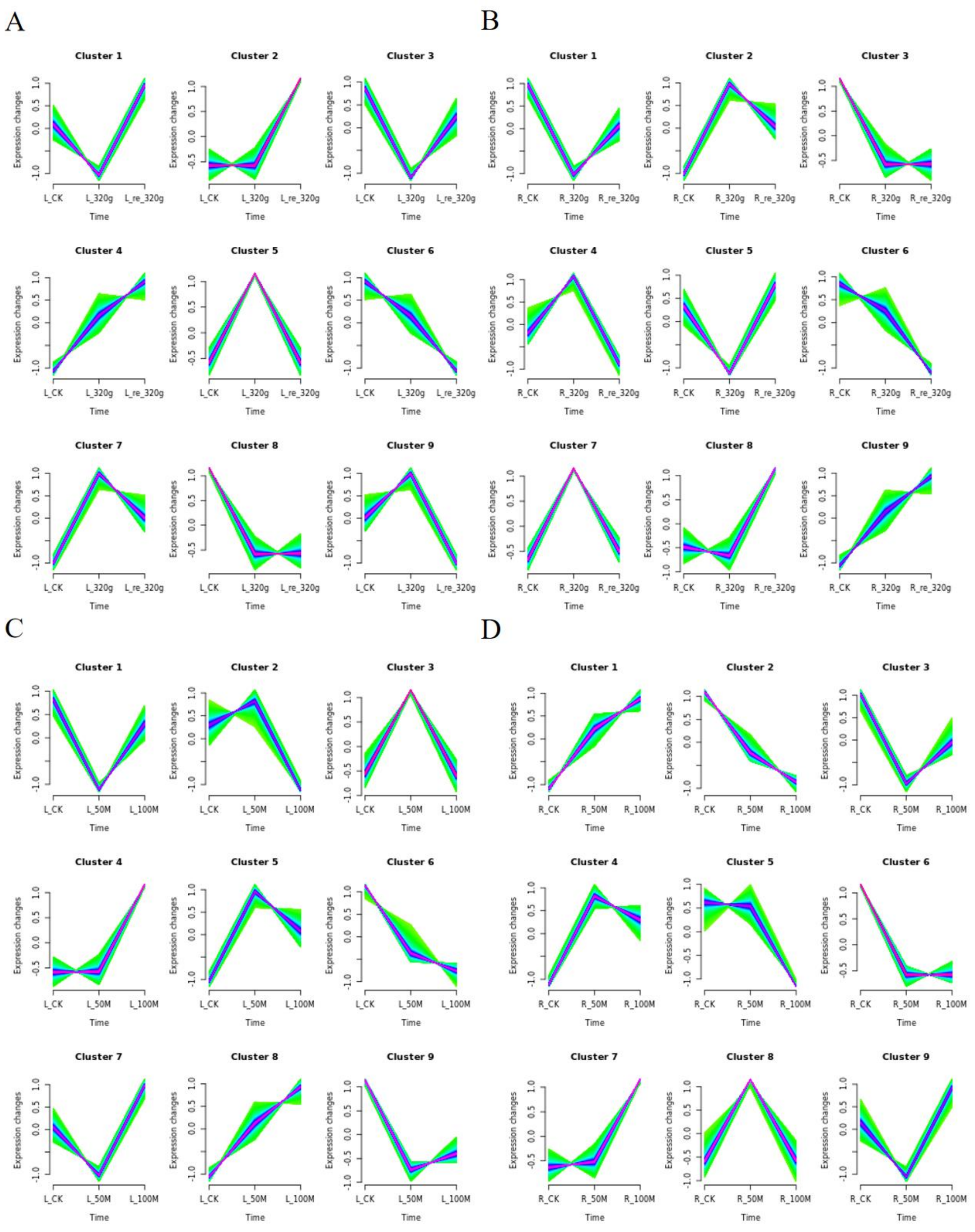

Figure S10. Changes in photosynthetic parameters of potted seedlings under drought (A,B) and salt (C,D) stress. L_CK, L_320g and L_re_320g are the leaves of control group, dried for 2 weeks and rehydrated for 24 hours, respectively; L_50M and L_100M are the leaves of plants treated with 50 mmol·L^{-1} and 100 mmol·L^{-1} salt for 2 weeks, respectively. R_CK, L_320g and R_re_320g are the leaves of the control group, dried for 2 weeks and rehydrated for 24 hours, respectively; R_50M and R_100M are the leaves of plants treated with 50 mmol·L^{-1} and 100 mmol·L^{-1} salt for 2 weeks, respectively.

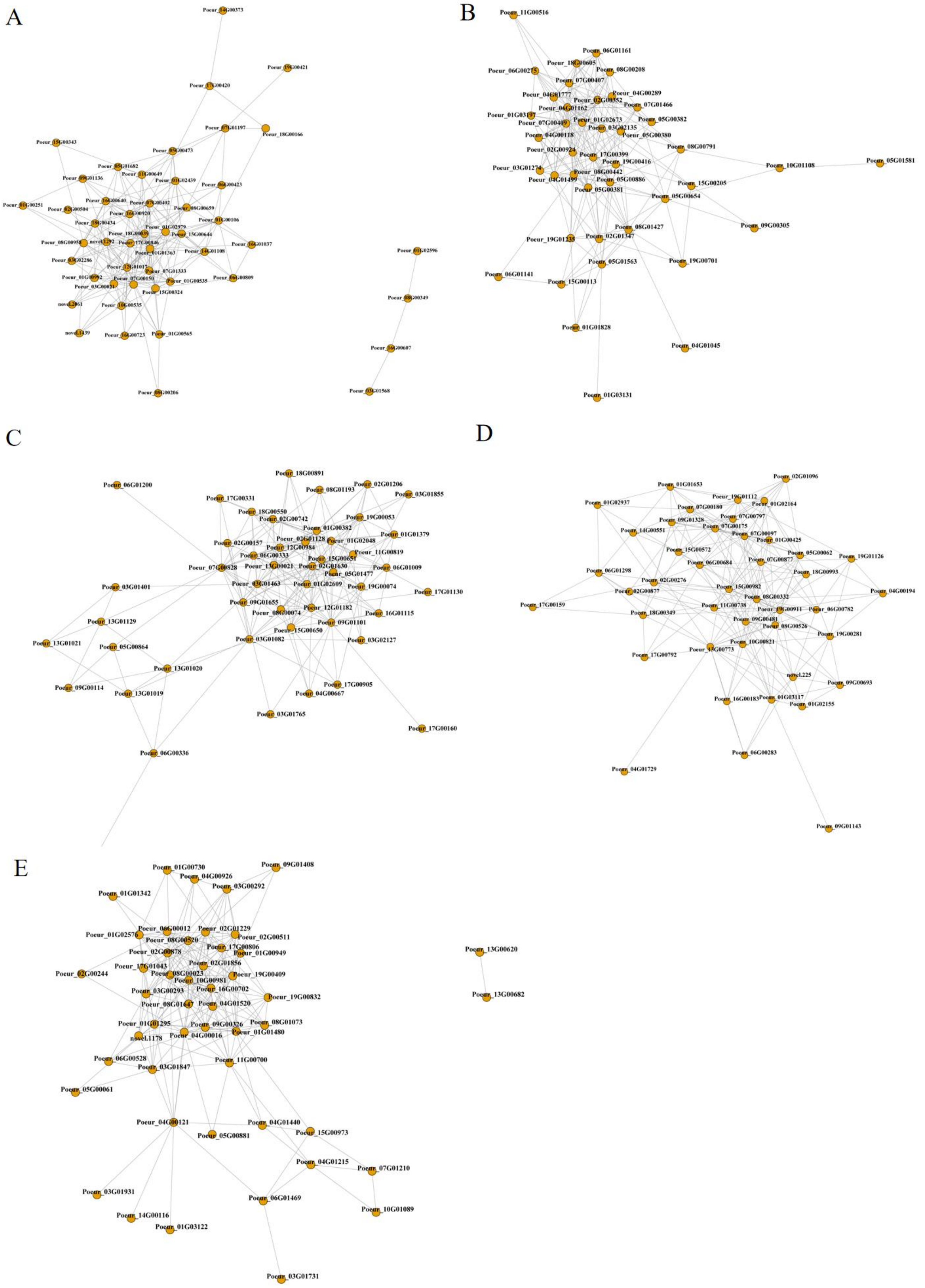

Figure S11. Co-expression network. A Royalblue hub genes. **B** Skyblue hub genes. **C** Darkmagenta hub genes. **D** Cyan hub genes. **E** Midnightblue hub genes.
